# *Heliconius* butterflies use wide-field landscape features, but not individual local landmarks, during spatial learning

**DOI:** 10.1098/rsos.241097

**Published:** 2024-11-06

**Authors:** P. A. Moura, M. Z. Cardoso, S. H. Montgomery

**Affiliations:** ^1^ Departamento de Ecologia, Universidade Federal do Rio Grande do Norte, Natal, Brazil; ^2^ Departamento de Ecologia, Instituto de Biologia, Universidade Federal do Rio de Janeiro, Rio de Janeiro, Brazil; ^3^ School of Biological Sciences, University of Bristol, Bristol, UK

**Keywords:** foraging ecology, *Heliconius *butterflies, insect navigation, landmark use, landscape cues, spatial learning

## Abstract

Spatial learning is vital in foraging ecology. Many hymenopteran insects are adept spatial foragers that rely on visual cues contained within broader wide-field scenes for central place foraging from a central nest. By contrast, for butterflies, which lack central nest sites, visual cue use during spatial foraging is less understood. *Heliconius* butterflies, however, exhibit stable nocturnal roosts, strong site fidelity and a sophisticated capacity for spatial navigation. This study furthers our understanding of *Heliconius* spatial learning by testing whether *H. erato* can associate a spatially informative visual cue with artificial feeders. We explored the relative importance of a visual local landmark compared with broader, wide-field visual cues, through experiments with (i) a fixed rewarded feeder with a local landmark; (ii) a mobile rewarded feeder with the landmark as the sole reliable cue; (iii) the same setup while blocking visual access to external landscape features. Our data suggest that *Heliconius* butterflies learn static feeder locations without relying on a local individual landmark. Instead, we suggest they integrate broader landscape and celestial cues. This suggests that *Heliconius* butterflies and central place foraging hymenopterans likely share similar visual navigation strategies, using wide-field, low-resolution views rather than focusing on specific individual landmarks.

## 1. Background

Foraging is central to any species’ survival and reproduction. Therefore, the ability to navigate through the environment by learning where and when to find food—known as spatial learning—is ecologically important to many species, including insects. Spatial learning refers to the formation of memories that help animals to distinguish a place by reference to its surroundings to relocate a particular position based on the relative orientation of the learner [1]. For spatial learning to occur, animals can exploit a diverse set of environmental features, such as the combination of terrestrial and celestial cues, to recall specific locations or routes [1–3].

Some insects, most prominently the social and parasitic Hymenoptera, are highly specialized to form reliable spatial memories that enable them to navigate to specific spatial goals. One key component of the base model for insect navigation, inspired largely by data from Hymenoptera, is the ‘view memory’ [4], which makes use of terrestrial cues from an insect’s surroundings and is based on memories of visual cues [3,5,6]. Formation of a view memory involves the storage of multiple views taken while an individual is facing or moving along a route toward a goal. During subsequent foraging events, the familiarity of that individual’s current view, based on how well their current visual input matches a previously stored view, helps guide movement [4,7]. One fundamental source of visual information that can be used by insects are visually salient cues that enable individuals to establish their location, known as landmarks [8]. The way landmarks are used by insects can vary [3,5,6,9] and includes (i) scene recognition when individuals are displaced from their home range, or nest, to another familiar location, as shown in bees [10–12], (ii) as a way of correcting paths when individuals are displaced from their stereotyped homebound paths, as demonstrated in ants [13–15], and (iii) as a way of pinpointing a hidden resource in the context of the wider panorama, as shown in solitary wasps [16]. The reliance on landmarks depends on the navigational information content of the habitat, with species in landmark-rich environments depending more on landmark cues and species in landmark-poor environments relying more on celestial compass for guidance [17,18]. Regardless, across insects, many species with notable vision-based navigational skills [5,6] use landmarks for directional guidance, and these cues help individuals return to important locations, such as a nest or a food source [13,14,16,19–24], a hovering station [25] or a safe place [26–28], encompassing different spatial scales. However, most insects do not rely solely on individual landmarks to the exclusion of the surrounding panorama [4,7,21]. They integrate a variety of features such as light, colours and motion parallax to compensate for the poor resolution of insects’ eyes [21].

Spatial learning in many hymenopteran insects is centred around a nest or a burrow, from and to which terrestrial and celestial visual cues are used to navigate [12–16,19,20]. However, reliance on food sources that are generally limited, but dependable and distributed in patches over a large territory, may also result in spatially faithful foraging behaviours, and this selection pressure may be independent of other traits associated with many Hymenoptera, such as nest building or social living. A particularly derived form of spatially faithful foraging is known as trap-lining, whereby individuals establish foraging routes along which specific patches or plants are visited in sequence with a high degree of regularity, resulting in an efficient route over time [29]. Trap-lining is particularly reported in hymenopterans, such as bumblebees [30–32], euglossine bees [33], and—to some extent—honey bees [34,35], where it is associated with allothetic foraging from a central nest (a navigation strategy centred on external references or cues). However, trap-lining is reported in some other invertebrates, including the Neotropical butterflies of the genus *Heliconius* (Heliconiinae, Nymphalidae) [36,37]. *Heliconius* therefore provide an interesting opportunity to study the use of visual cues during spatial learning in an insect that lack a pronounced central nest, in a phylogenetically distant lineage of insects.

*Heliconius* traplines are centred on a limited home range of 100 m^2^ to 1 km^2^ [38,39] with a high degree of site fidelity [40]. Within these home ranges individuals return to a restricted number of roosting locations at night [38,41–43], from which they start their foraging routes the following day, providing the context for allothetic foraging. *Heliconius* butterflies also have a novel dietary adaptation, active pollen feeding, which is thought to be unique among butterflies and may explain the evolution of trap-lining in this genus [44,45]. They actively collect and digest pollen grains from a restricted set of plants, from which they extract essential amino acids [46] critical to their increased longevity [36], delayed reproductive senescence [47], and chemical communication and toxicity [48–50]. The reliability of pollen sources, particularly perennial vines that flower year-round [51], contrasts with their generally limited and patchily distributed abundance [44]. This likely favours the evolution of trap-lining behaviour as the optimal foraging strategy, with the value of long-term memories further enhanced by the elongated lifespan of *Heliconius* [47].

Together, these behaviours suggest a sophisticated capacity for spatial navigation in *Heliconius*, which is hypothesized to have evolved the capacity to learn visual landmarks [36,37,42,52], similar to behaviours observed in bumblebees [29,32]. Notably, *Heliconius* have derived neural morphologies that may be associated with this behaviour, in particular, well-developed neural structures key to associative learning and attention in insects, the mushroom bodies [53–55]. *Heliconius* mushroom bodies are the largest among all species of Lepidoptera and show evidence of specialization towards visual processing [56,57]. Mushroom bodies are crucial for view-based navigation in cockroaches [26], ants [58,59] and *Drosophila* [60], allowing them to recognize and remember learned visual scenes and use them to navigate to food sources and back to the nest. This type of navigation is acquired through experience and learning, with inputs from pre-motor areas of the brain involved in processing motor commands [61], contrasting with innate visual behaviour, which includes the processing of visual information in more peripheral circuits [59]. Models of mushroom body function suggest that larger circuits should support an ability to store more images of landscape features [62], further supporting a hypothesized link between *Heliconius* foraging, mushroom body expansion and memory capacity.

Although it has been speculated that *Heliconius* butterflies make use of landmarks within the landscape to pinpoint the location of resources within their home range [52], no studies have specifically tested the hypothesis of visual landmark use in *Heliconius*, or examined the scale at which visual cues are used. While Hymenoptera studies suggest that these species do not have the ability to isolate landmarks from the rest of a scene [21], a recent study on non-migratory monarch butterflies (*Danaus plexippus*) suggested that these butterflies are able to learn to track a local individual landmark closely associated with a rewarded feeder when both are moved within a cage [63], providing an exception to the rule applied to most studied insects. The only study currently available for *Heliconius* is from the 1930s and suggested that individual *Heliconius* that regularly returned to a specific nocturnal roost location still gathered at that roost site even after branches in the roost were experimentally displaced to a new location, or replaced by new branches [42]. This led to the long-held view that roost sites are identified by general visual cues, rather than by olfactory cues [42].

In the present study, we test whether individuals of *H. erato* can learn to associate a nearby, spatially informative object (a ‘local individual landmark’) with a food source in free-flying experiments, or if they learn spatial information using broader, more inclusive wide-field visual cues. For this purpose, we designed a series of experiments that progressively assess the relative importance of local and broader features by training individual butterflies to visit one of four artificial feeders within a cage while manipulating the reliability of visual cues provided by the local individual landmark. Our results concur with hypothesized patterns, suggesting that *Heliconius* likely forsake local landmarks in favour of wider landscape features in combination with celestial cues such as the sun azimuth and polarized light patterns.

## 2. Methods

### 2.1. Experimental subjects

Study subjects originated from first-generation insectary-reared stock populations of *H. erato phyllis*, descended from wild-caught individuals collected in Mata do Jiqui, Natal, Brazil (5°55′47′ S 35°11′06′ W). We maintained stock populations in ambient conditions in a partially shaded outdoor cage (approx. 3 × 3 × 2.5 m). Stock butterflies had access to two local host plant species (*Passiflora misera* and *P. galbana*) and artificial feeders containing approximately 20% sugar solution with additional bee-pollen supplement as a source of amino acids. For all experiments, butterflies were individually labelled with unique IDs using a nontoxic ink mark on the ventral side of their forewings. Experimental butterflies participated in only one of the experiments and were trained to feed on artificial feeders made from red foam sheets and yellow plastic straws. Rewarded feeders contained a 20% sugar solution with an additional bee-pollen supplement, while unrewarded feeders were empty.

### 2.2. Experimental design

The study design is based on protocols previously used to assay landmark use in spatial memory tasks in hummingbirds [64,65]—a vertebrate group that trap-lines and uses local individual landmarks to navigate—and wasps [66]. The experiments were performed within an approximately 3 × 3.5 × 2.5 m cage made of wood and green mesh netting, enabling individuals to experience surrounding ambient light conditions and landscape cues. Within the cage we included four identical feeding stations (feeders), each composed of four 4 cm artificial flowers placed on a 14 cm circle made from green foam sheets affixed to the top of 1 m tall stands. Feeders were placed on the ground 2 m apart in a square-shaped manner (electronic supplementary material, figure S1). Only one of the feeders was rewarded while the other three were unrewarded. As a local individual landmark within the cage, we constructed a visually prominent, 60-cm-tall, black-striped, green cylinder (foam sheet and black duct tape) affixed to the top of a 1 m tall brown PVC pipe, which we positioned adjacent to a feeder (40 cm away and at a 45^o^ angle to the left of the feeder; electronic supplementary material, figure S1), depending on the experiment and group. Green was chosen for the local individual landmark to mimic vegetation, and because *Heliconius* has good perception of this colour [67]. Indeed, the landmark was acknowledged by the butterflies during the experiment either by inspecting it (front facing circling behaviour) or landing on it.

Following a previous protocol that successfully induced learned spatial memories in *Heliconius* [39]*,* we trained and tested all butterflies individually for 10 min. All trials were run between 0800 h and 1400 h. To ensure motivation during the experiment, butterflies were not fed outside of training, or between the training and test trials. Butterflies were released individually into the cage through a small door on one side of the cage during training and testing. All individuals visited all four feeders across the training period.

Within this general framework we performed three sets of experiments: (i) Experiment 1 tests whether *Heliconius* can learn the position of a rewarded feeder associated with a landmark, both of which were fixed in place within a transparent-walled cage; (ii) Experiment 2 tests whether *Heliconius* can learn an association between a rewarded feeder and a mobile landmark when both were randomly positioned together, such that within a transparent-walled cage the only reliable cue was the position of the landmark; (iii) Experiment 3 tests whether *Heliconius* can learn the position of a rewarded feeder in an environment without surrounding landscape features.

### 2.3. Experiment 1: fixed local landmark

**Figure 1.**
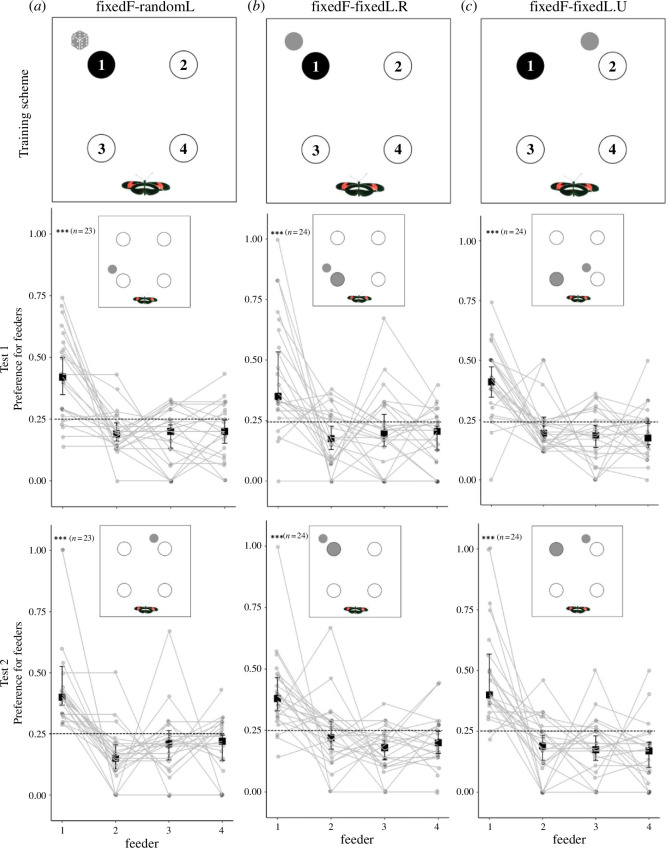
Fixed local landmark learning experiment. Columns: (*a*) fixed rewarded feeder and landmark with randomized positions (fixedF-randomL); (*b*) fixed rewarded feeder and adjacent fixed landmark (fixedF-fixedL.R); (*c*) fixed rewarded feeder and fixed landmark next to an unrewarded feeder (fixedF-fixedL.U). Rows: *Training scheme*. Black circles represent the rewarded feeder, which was fixed in the same position for all groups, while unfilled circles represent unrewarded feeders. Grey dice illustrates that the landmark had randomized positions in the control group (*a*). Small grey circles illustrate the landmark, which was fixed in a particular position (*b,c*). Feeders’ names correspond to quadrants (1 to 4). *Test 1 and Test 2*. Relative number of feeding attempts on all feeders in the trained preference test in Experiment 1. A feeding attempt corresponds to a butterfly landing on a flower and probing it with the proboscis. Larger grey circles represent the expected preferred feeder for experimental groups, while unfilled circles represent unrewarded feeders. Feeder 1 is here annotated as the rewarded feeder regardless of its position in the training phases. Small grey circles illustrate the landmark, which was fixed in position during the test for all groups. The dotted line shows the random expectation of each feeder. Squares and whiskers represent medians of individuals’ preference ± 95% CI. ***p <0.001.

We first tested whether *Heliconius* use a local landmark to learn the position of a rewarded feeder, both of which were fixed in place. The experiments comprised three different groups, all of which had the rewarded feeder fixed in the same position for the entirety of the experiment: (i) an experimental group trained with a fixed reward position and a fixed, adjacent landmark at the rewarded feeder (fixedF-fixedL.R, figure 1*b*); (i) an experimental group trained with a fixed reward position and a fixed, distant landmark next to a neighbouring, unrewarded feeder (fixedF-fixedL.U, figure 1*c*); (iii) a control group, where the position of the landmark was randomized between trials, such that it was not associated with any feeder in particular (fixedF-randomL, figure 1*a*), while the rewarded feeder remained in a fixed position.

For all groups, the experiment consisted of five phases. (i) During pre-training, naive individuals were fed on white feeders in a separate cage for 8 h (0700 h to 1500 h) to get accustomed to artificial feeders. The feeders were then removed to prevent feeding until the following day. (ii) In the first training phase, all groups were trained to feed on a fixed rewarded feeder for 4 days (figure 1, Training). In this training phase, the position of the landmark was either fixed (figure 1*b,c*) or randomized according to group membership (as described above) (figure 1*a*). Butterflies were released individually into the cage through a small door on one side of the cage. (iii) After 4 days of training, we performed the first trained preference test (figure 1, Test 1). In this test (Test 1), we sought to determine whether an individual had learned an association between the rewarded feeder and the landmark. To do so, for all groups, we fixed the landmark in a new position (figure 1, small grey circle) such that butterflies which had learned to associate the feeder and landmark would preferentially feed on feeder 3 (illustrated by the larger grey circle in figure 1). If, instead, they learned the position of the rewarded feeder independently of the landmark, they would preferentially feed on position 1 (figure 1, as indicated by the black circles). For the control group, we expected that butterflies would show no preference for any feeder and would visit all feeders equally. We recorded individual preference (number of feeding attempts) using clean, empty feeders. (iv) We subsequently extended the training phase for 2 days with the landmark and rewarded feeders positioned as per an individual’s initial training period, before moving to the final trained preference test. (v) In a second testing phase (Test 2), landmarks were positioned in the same place as in the training phase (figure 1, Test 2). This test therefore acts as a general test for learning the position of the feeder, with or without learning the association with the landmark. For the experimental groups, we expected that butterflies would attempt to feed more often on the feeder 1, illustrated by the larger grey circle in figure 1*b,c*. For the control group, we expected that butterflies would show no preference for any feeder and would equally feed on all feeders (figure 1*a*).

### 2.4. Experiment 2: randomized landmark position

**Figure 2.**
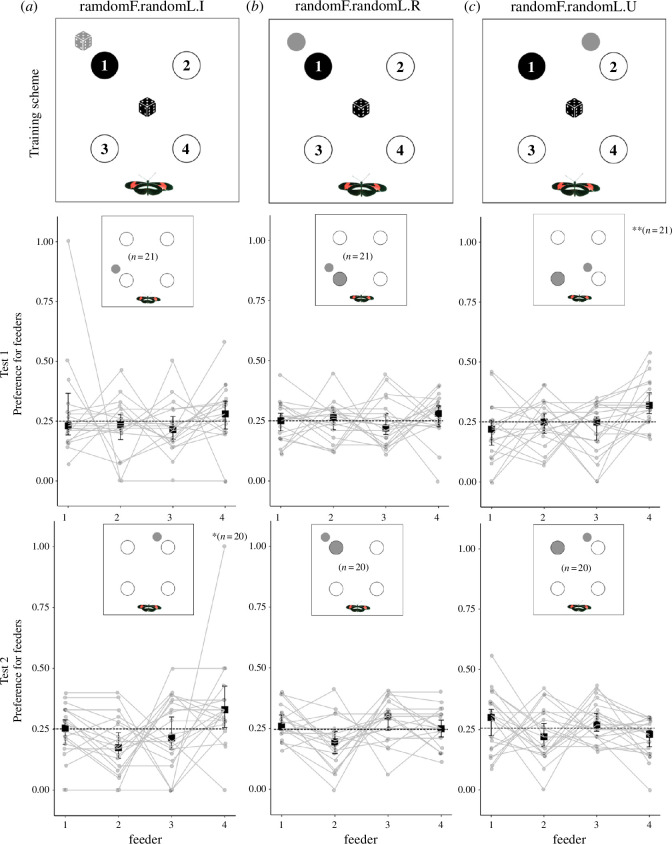
Randomized landmark position learning experiment. Columns: (*a*) independently randomized rewarded feeder and landmark (randomF-randomL.I); (*b*) random rewarded feeder and adjacent random landmark (randomF-randomL.R); (*c*) random rewarded feeder and adjacent random landmark next to an unrewarded feeder (randomF-randomL.U). Rows: *Training scheme*. Black circles represent the rewarded feeder, while unfilled circles represent unrewarded feeders. Grey dice illustrates that the landmark had independent randomized positions in the control group (*a*). Small grey circles illustrate the landmark, which moved concurrently with the rewarded feeder (*b,c*). Black dice illustrates that the rewarded feeders had randomized positions (*a,b,c*). Feeders’ names correspond to quadrants (1 to 4). *Test 1* and *Test 2*. Relative number of feeding attempts on all feeders in the trained preference test in Experiment 2. A feeding attempt corresponds to a butterfly landing on a flower and probing it with the proboscis. Squares and whiskers represent medians of individuals’ preference ± 95% CI. **p < 0.01, *p < 0.05.

In Experiment 2, we tested whether *Heliconius* can associate the presence of a reward with a landmark when the position of both were randomized but moved in a predictable, consistent partnership. In this experimental setting, the only reliable local visual cue of the rewarded feeder position would be the landmark. The experiments comprised three different groups: (i) an experimental group in which the landmark was adjacent to the rewarded feeder, and the position of both changed concurrently (randomF-randomL.R, figure 2*b*); (ii) an experimental group in which the landmark was adjacent to an unrewarded feeder, but the position of both the rewarded feeder and landmark still changed concurrently (randomF-randomL.U, figure 2*c*); (iii) a control group in which the position of the rewarded feeder and landmark were randomized independently (randomF-randomL.I, figure 2*a*).

The experiment consisted of five phases. (i) Pre-training as in Experiment 1. (ii) A first training phase of 4 days, both the position of the rewarded feeder and landmark differed each day throughout the training phase for all groups (figure 2). As described above, the landmark moved concurrently with the rewarded feeder in experimental groups (i) and (ii); while for the control group (figure 2*a*), both the landmark and the rewarded feeder moved independently each day during the training phase, meaning that the landmark was most often not associated with the rewarded feeder. (iii) Following 4 days for training, we performed the first trained preference test using clean, empty feeders, and measuring individual feeding attempts (Test 1) where the landmark was fixed in a random position (figure 2, Test 1). For the experimental groups, if they learned the association between landmark and rewarded feeder, we expected that butterflies would land more often on the feeder associated with the landmark (figure 2, larger grey circle). For the control group, we expected that butterflies would show no preference for any feeder and would land with equal frequency on all feeders. (iv) We subsequently extended the training phase for 2 days before moving to the final trained preference test. (v) In the final preference test (Test 2), we again randomized the position of rewarded feeder and landmark, as per their group descriptions, and recorded individual preferences as described above, using clean, empty feeders (figure 2).

### 2.5. Experiment 3: removing external landscape cues

The previous experiments permitted the use of visual cues external to the cage walls, which are constructed of transparent mesh, in addition to the landmark inside the cage. For the final experiment, we sought to remove these cues by covering the outer surface of the cage walls in opaque black sheets. The cage ceiling remained uncovered (excluding the original cage mesh net) to allow natural light in to maintain illuminance levels and prevent overheating. From the position of the feeders, no visual landscape cues were visible through the ceiling of the cage to a human experimenter, however, other solar cues would have been available to the butterflies.

**Figure 3.**
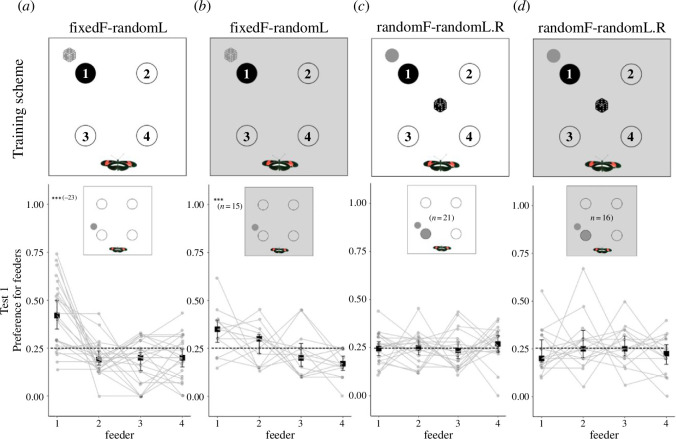
Removal of external cues. Columns: (*a*) group fixedF-randomL from Experiment 1; (*b*) dark fixedF-randomL; (*c*) group randomF-randomL.R from Experiment 2; (*d*) dark randomF-randomL.R. Rows: *Training***.** Same as figures 1 and 2. *Test 1*. Relative number of feeding attempts on all feeders in the trained preference test in Experiment 2. A feeding attempt corresponds to a butterfly landing on a flower and probing it with the proboscis. Squares and whiskers represent means of individuals’ preference ± 95% CI. ***p < 0.001.

In Experiment 3, we repeated two previous experiments with fresh, naive butterflies, to see whether the absence of external cues would change the butterflies’ perception of the landmark: (i) to test whether *Heliconius* can still learn the position of a rewarded feeder without the surrounding visual scene, we repeated the test (Experiment 1) with a fixed rewarded feeder position and a randomized local landmark position (fixedF-randomL; figure 3*b*); (ii) to test if *Heliconius* rely more on local landmark cues in the absence of a wider visual scene, we repeated the experiment (Experiment 2) in which the positions of the rewarded feeder and an adjacent landmark were moved together concurrently (randomF-randomL.R; figure 3*d*). For each group, the experiment was run identically to the equivalent experiment described above, but within the cage with opaque walls.

### 2.6. Statistical analysis

In all trials feeding attempts were recorded as the butterfly landing on a particular feeder, extending and probing a flower with its proboscis. If a butterfly made several consecutive probing attempts on the same flower without flying away, we recorded as a single feeding attempt. Feeder preference is therefore the proportion of feeding attempts made to each feeder. Preference data were analysed using generalized linear mixed-effects models (GLMMs) in R [68] with the glmer function from the package lme4 v 1.1–31 [69]. All models were fitted with binomial distributions and included an individual-level random effect to account for overdispersion. Models were assessed for compliance with statistical assumptions using the R package DHARMa v 0.4.6 7 [70]. For all experiments, we examined whether there were differences in relative preference for the four feeders per group in the trained preference tests, with the response variable ‘preference for feeders’ (relative number of feeding attempts on each feeder, here referred to as feeding attempts), and fixed factors ‘group’ and ‘feeder’ (1 to 4). For each experiment, the fixed factor ‘group’ was: fixedF-randomL, fixedF-fixedL.R, fixedF-fixedL.U (Experiment 1); randomF-randomL.I, randomF-randomL.R, randomF-randomL.U (Experiment 2); and randomF-randomL.R, fixedF-randomL (Experiment 3). All experiments included male and female individuals.

## 3. Results

### 3.1. *Heliconius* butterflies do not need a local individual landmark to learn the position of a reliable food source

In Experiment 1, if individuals used the position of the landmark as an associated cue for identifying rewarded feeders during training, we expected individuals from both experimental groups to land more often on the feeder position indicated by the larger grey circles in figure 1*b,c*. By contrast, we expected individuals from the control group to randomly choose among all feeders in both preference tests (figure 1*a*), as the landmark provided no reliable information. However, we found no difference in feeder preference between control and experimental groups (Test 1: *χ*^2^ = 2.41, d.f. = 2, *n* = 71, *p* = 0.30; Test 2: *χ*^2^ = 2.20, d.f. = 2, *n* = 71, *p* = 0.33), showing individuals from all groups, including the control group, landed more often on the feeder that was rewarded during the training phase (Test 1: *χ*^2^ = 106.86, d.f. = 1, *n* = 71, *p *< 0.001; Test 2: *χ*^2^ = 104.82, d.f. = 1, *n* = 71, *p* < 0.001). In the control group, we again found that individuals highly preferred the feeder that presented the reward during training for both first (*χ*^2^ = 33.48, d.f. = 1, *n* = 23, *p* < 0.001; figure 1*a*, Test 1) and final (*χ*^2^ = 22.54, d.f. = 1, *n* = 23, *p* < 0.001; figure 1*a*, Test 2) trained preference tests.

The same pattern was observed for both experimental groups. In the group with a fixed rewarded feeder with an adjacent fixed landmark (fixedF-fixedL.R), individuals highly preferred the feeder that presented the reward during training for both first (*χ*^2^ = 23.18, d.f. = 1, *n* = 24, *p* < 0.001; figure 1*b*, Test 1) and final (*χ*^2^ = 38.05, d.f. = 1, *n* = 24, *p* < 0.001; figure 1*b*, Test 2) trained preference tests. In the second experimental group, where the rewarded feeder was fixed with a fixed landmark next to an unrewarded feeder (fixedF-fixedL.U), individuals also highly preferred the feeder that presented the reward during training for both first (*χ*^2^ = 52.61, d.f. = 1, *n* = 24, *p* <0.001; figure 1*c*, Test 1) and final (*χ*^2^ = 46.02, d.f. = 1, *n* = 24, *p *< 0.001; figure 1*c*, Test 2) trained preference tests. The consistency between Tests 1 and 2 occurs despite the local landmark being displaced in Test 1, but not Test 2, relative to the position during training (*z* = −0.19, *n* = 71, *p* = 0.85). These results show *Heliconius* can learn a fixed feeder position, but do not preferentially do so using a local individual visual landmark.

### 3.2. *Heliconius* butterflies did not associate a food source to an adjacent individual landmark

In Experiment 2, we sought to test if *Heliconius* will switch to using a local visual cue provided by an individual landmark when other visual cues are unreliable. We found no difference in feeder position preference between control and experimental groups in Test 1 (*χ*^2^ = 2.87, d.f. = 2, *n* = 63, *p* = 0.24) but only in Test 2 (*χ*^2^ = 6.34, d.f. = 2, *n* = 61, *p* < 0.05). In the control group (randomF-randomL.I), we expected that feeding attempts would be random as the local landmark was not a reliable indicator of the position of the rewarded feeder. This expectation was met in Test 1 (figure 2*a*, Test 1: *χ*^2^ = 4.30, d.f. = 1, *n* = 21, *p* = 0.10) but not in Test 2 (figure 2*a*, Test 2: *χ*^2^ = 5.71, d.f. = 1, *n* = 20, *p *< 0.05), with feeder 4 being highly preferred despite no association with the landmark, suggesting a positional bias unrelated to the rewarded feeder. In the group randomF-randomL.R, where the landmark was always adjacent to the rewarded feeder during training, we did not observe any preference for any of the feeders (figure 2*b*, Test 1: *χ*^2^ = 1.09, d.f. = 1, *n* = 21, *p* = 0.30; figure 2*b*, Test 2: *χ*^2^ = 0.13, d.f. = 1, *n* = 20, *p* = 0.72;). While in the group randomF-randomL.U, where the landmark was a more distant, but still reliable cue, this lack of preference was also observed in the second preference test (figure 2*c*, Test 2: *χ*^2^ = 1.19, d.f. = 1, *n* = 21, *p* = 0.27;) but in the first preference test we again detect an incorrect positional bias towards feeder 4 (figure 2*c*, Test 1: *χ*^2^ = 10.32, d.f. = 1, *n* = 21, *p* < 0.01). These results show *Heliconius* do not switch to using cues from a local individual landmark to direct foraging preference, even when that landmark offers the sole reliable positional cue within the cage.

### 3.3. *Heliconius* butterflies learn positional information but do not use an informative individual landmark even when the external visual scene is simplified

Given that individuals in the control group in Experiment 1 (fixedF-randomL) had learned the position of the food despite a randomly positioned local landmark, we repeated the same experiment with opaque walls to remove surrounding landscape features. Repeating this experiment with opaque cage walls, we still found evidence that *Heliconius* learn the location of the fixed rewarded feeder (*χ*^2^ = 19.18, d.f. = 1, *n* = 15, *p* < 0.001; figure 3*b*). Comparing the results from data obtained with opaque/transparent cage walls, we observed no difference in feeder preference, although there was a trend towards improved performance when landscape cues were available (*χ*^2^ = 3.16, d.f. = 1, *n* = 38, *p* = 0.07). We next repeated the experiment in which the position of the rewarded feeder and an adjacent local landmark were randomized in tandem during training, to test whether *Heliconius* switch to reliance on local landmarks when deprived of broader visual scenes. Comparing the results from individuals assayed with opaque/transparent cage walls, we observed no difference in feeder preference (*χ*^2^ = 0.27, d.f. = 1, *n* = 36, *p* = 0.60) (feeder 3, *t* = −0.82, *n* = 17, *p* = 0.42), and in the opaque cage group we again did not observe any feeder preference (*χ*^2^ = 0.15, d.f. = 1, *n* = 16, *p* = 0.70; figure 3*d*), implying that even when broader cues are unavailable, *Heliconius* do not switch to using local landmark cues.

## 4. Discussion

Some insect species, most prominently the social and parasitic Hymenoptera, form reliable spatial memories that enable them to navigate to specific spatial goals. Like bumblebees [30–32], euglossine bees [33] and honeybees [34,35], *Heliconius* butterflies present trap-lining behaviour likely using visual features of their environment. Here, we tested whether *Heliconius* butterflies were able to learn the spatial location of a rewarded artificial feeder by using visual cues across three different experimental conditions. Our results suggest that these butterflies likely use a combination of landscape visual cues and celestial compass cues to find the location of a dependable food reward in a series of spatial learning tasks, but do not extract information from individual objects adjacent to rewarding flowers, suggesting they do not rely on individual landmarks. Although it is not clear what exact cues these butterflies used, our data demonstrate that local landmarks close to the goal were not used in these tasks.

When individual butterflies were trained in an arena with a dependable food source (fixed rewarded feeder), the position of an adjacent visual cue (black-striped, green cylinder) provided reliable and informative positional cues, but was not utilized to guide foraging behaviour (Experiment 1). Learning the position of the rewarded feeder occurred despite the presence of the local individual landmark, which is consistent with anecdotal evidence that more broad landscape features are important for *Heliconius* foraging [42], as well as more recent findings that show that *Heliconius* have positional memory in foraging contexts at different spatial scales in semi-natural conditions [39]. The exact visual cues used by individual butterflies foraging in the wild could range from the image of the floral resources themselves to locally associated visual cues, or more distant landscape cues. Here, by using visually consistent feeders and local individual landmarks, we suggest that spatially distant cues may be particularly relevant for spatial learning in *Heliconius* butterflies.

In the follow-up experiment (Experiment 2), we aimed to test whether *Heliconius* could use the local individual landmark to learn the position of a moving food source (feeder with randomized position) when this became the only reliable information source in the assay. In this scenario, since we used an individual landmark (black-striped, green cylinder) that moved concurrently with the food source, we expected that, if *Heliconius* extract information from local, individual landmarks, these butterflies would learn the association between the reward and the local individual landmark. However, we did not observe any evidence that the butterflies were able to learn this association. By contrast, the only two instances in which butterflies did show a preference for one of the four feeders (randomF-randomL.I; randomF-radomL.U) occurred when the feeders were not associated with the local individual landmark. In both cases, the feeder of choice was the last feeder presented with the reward during training, suggesting that when faced with a spatially unreliable resource, individuals deferred to the position of the last rewarded feeder rather than the local landmark, despite the reliability of this cue.

In the final experiment, butterflies were trained in an arena where the complexity of external landscape cues had been reduced (walls covered in opaque black sheets; Experiment 3). We again found evidence that *Heliconius* were able to learn the position of the rewarded feeder, which again occurred without reliance on an associated local individual landmark. In this case, in the absence of external landscape cues, individual butterflies presumably had greater reliance on celestial cues when navigating in the right direction within the arena. While comparisons between the fixed feeder experiments with and without the greater availability of external landscape cues were not significant, performance was generally higher when they were available. When reduced, butterflies showed a preference for the rewarded feeder but also the feeder facing the same side of the arena as the rewarded feeder, suggesting the accuracy of the learned position was more general, with butterflies possibly learning the feeder configuration and using celestial compass cues to find the correct feeder. While we cannot rule out other cues related to subtle differences in the cage walls, recent visual acuity data for *Heliconius erato* [71] would suggest this level of attentive perception is perhaps unlikely. Nevertheless, the geometrical structure of the cage, emphasized by the contrast between the black walls and the sky, coupled with celestial cues—such as polarized light, light gradients and chromatic gradients—and differential lighting of the cage walls due to the sun’s changing position during the experiment, likely provided enough conspicuous information for the butterflies to return to the same feeder [7,21,22,72]. However, since we were unable to control for all these factors, we cannot determine the relative importance of each cue in this experiment.

In other insect systems which display allothetic foraging, individuals simultaneously encode a combination of multiple landmarks—either local or distributed across the landscape—and celestial cues such as the sun azimuth and polarized light patterns [21–24,73–75]. The weight given to each of these cues depends on their reliability and stability in the species’ habitat [17,18]. Our results indicate that for *Heliconius* butterflies, local individual landmarks are likely not used as an aid to find a food source and, rather, wide-field landscape information is more important in conjunction with celestial cues.

As *Heliconius* butterflies display site fidelity by returning to their site of origin after displacement [40] and present a limited home range in which they have established their daily foraging routes and return to specific roosting locations in the evening, we expected that these butterflies would rely on learned visual cues to find the location of important resources. While *Heliconius* are unique in the extent to which they have specialized their derived spatial foraging behaviour, the use of spatial information and formation of spatial memories is not restricted to trap-lining species. Indeed, a recent study showed that (non-migratory) monarch butterflies (*Danaus plexippus*) can learn the location of a food source using spatial cues associated with the arrangement of multiple landmarks within a small cage [63]. *Danaus* are also able to learn to track salient local individual landmarks closely associated with a rewarded feeder when both are moved within a cage [63], in a way that our experiments suggest *Heliconius* do not. These results may suggest that *Danaus* may be more reliant on short range spatial cues than *Heliconius*, which instead is specialized to maximizing the formation of long-range spatial memories. Differences in experimental design, such as the distance between feeders and their orientation, could also explain some of these patterns, but we suggest more comparative learning assays across lepidopterans with divergent foraging ecologies would help characterize specializations in spatial memory. Indeed, these results suggest a form of spatial learning ability may be more widespread across lepidopterans than appreciated, which may have facilitated the initial transition of pollen feeding and trap line foraging in *Heliconius*, with possible subsequent refinement of the cues used in navigation, and an expansion of the number of visual scenes capable of being stored within the mushroom body circuitry [62].

## 5. Conclusions

Our data strongly suggest that *Heliconius* butterflies do not rely on local learned visual cues associated with individual landmarks to pinpoint the location of a food source but most likely rely on the coupling of wide-field visual scenes and celestial cues. We hypothesize that in the visually complex environments in which *Heliconius* occur, general landscape cues coupled with celestial cues may provide a more accurate and temporally stable strategy for identifying patchily distributed pollen sources where large-scale environmental features are more stable than local vegetation. Our study suggests that *Heliconius* butterflies have likely developed a visual navigation system similar to that of central place foraging hymenopterans, relying on wide-field, low-resolution views instead of focusing on individual objects, even when the latter are the only indicators of a food source.

## Data Availability

Data and R scripts are available at FigShare [76].
